# Pulmonary *Nocardia* infection in a child with idiopathic pulmonary hemosiderosis

**DOI:** 10.1186/s12890-021-01544-0

**Published:** 2021-05-29

**Authors:** Lu Qin, Fei-Zhou Zhang, Tong-Yu Yang, Xiao-Fen Tao, Lan-Fang Tang

**Affiliations:** Department of Pulmonology, Children’s Hospital of Zhejiang University School of Medicine, 3333 Binsheng Road, Hangzhou, 310051 China

**Keywords:** Pulmonary hemosiderosis, Idiopathic, *Nocardia*, Corticosteroid, Hemosiderin-laden macrophages

## Abstract

**Background:**

Idiopathic pulmonary hemosiderosis (IPH) encompasses a rare and agnogenic group of diffuse alveolar capillary hemorrhagic diseases. Corticosteroid treatment is the globally preferred therapeutic strategy for IPH; however, it can cause immunodeficiency. *Nocardia* infection often occurs in immunocompromised patients and primarily involves the pleura and lungs. Herein, we describe a case of pediatric pulmonary Nocardia infection after the corticosteroid treatment of IPH.

**Case presentation:**

A 7-year-old girl presented with chief complaints of pale complexion persisting for 1 year and a cough for 20 days. Abundant hemosiderin-laden macrophages were detected in the gastric juice, which supported the diagnosis of IPH. Uninterrupted doses of corticosteroids were administered during the last hospitalization. After nearly 2 months of corticosteroids therapy, the patient began to cough and produce a purulent sputum. Next-generation sequencing of the bronchoalveolar lavage fluid revealed *Nocardia abscessus* (*N. abscessus*) DNA. Linezolid was administered with good response, and the patient was discharged after 18 days of hospitalization. Her symptoms and pulmonary lesions had recovered, and the IPH appeared to be well-controlled with low dose of corticosteroids in follow-up.

**Conclusions:**

Nocardia infection should be considered in the differential diagnoses for IPH patients receiving corticosteroid therapy, especially in patients with poor response to conventional empirical antibiotic therapy. Next-generation sequencing of bronchoalveolar lavage fluid may be used to quickly identify the Nocardia. Sulfonamides or linezolid are effective for pediatric pulmonary Nocardia infection.

## Background

Idiopathic pulmonary hemosiderosis (IPH) is characterized by the copious deposition of hemosiderin-laden macrophages in the lung. IPH has an incidence of approximately 0.24–1.23 per million people and predominantly affects children [1]. However, the exact pathogenetic mechanism of IPH remains unclear. Hemoptysis, lung infiltration, and iron-deficiency anemia (IDA) is the generally recognized triad of symptoms in pediatric patients with IPH, and the typical earliest clinical manifestation in children with IPH is unexplained anemia [2]. Corticosteroid (CS) therapy is the treatment of choice for IPH [3]. *Nocardia* is an aerobic actinomycete and is not part of the normal flora of the human body. Pulmonary nocardiosis is a well-described infectious disease in immunosuppressed patients (ISPs) and in immunocompetent patients (ICPs) [4]. This disease is similar to acute bacterial pneumonia, and approximately 90% of cases are caused by *Nocardia stellate* [5]. Herein, we describe a pediatric case with IPH who developed a secondary N. abscessus infection after CS treatment.

## Case presentation

**Fig. 1:**
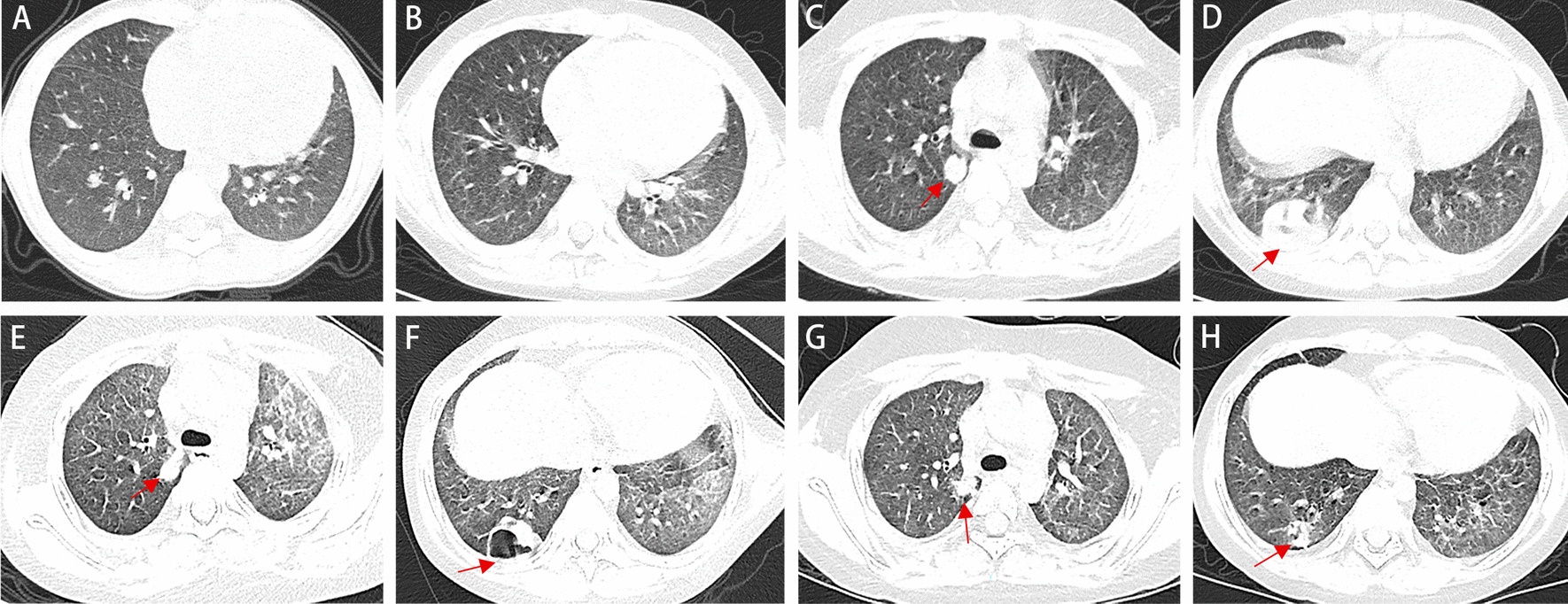
Changes in chest imaging of the patient. **a** Diffuse fine granular shadows were observed in both lungs (2 months before admission). **b** Diffuse granular shadows in both lungs and round nodular high-density shadows in the right lung (14 days before admission). **c**–**d** The transmittance of the disease into the bilateral lung decreased unevenly, and there were still fine particles in the lungs. The right lung contained scattered, circular lesions, and the boundaries were clear, the arrors showed two large lesions, the larger is about 30 × 42 × 27 mm (on the day of admission). **e**, **f** The lesions in the lower lobe of the right lung were improved, but cavity formation, new lesions in the left lung, and bilateral pleural thickening were observed (on the 9th day of admission). **g**, **h** The lesions in the inferior lobe of right lung with cavity were significantly reduced, and the changes of both lungs were improved (on the 16th day of discharge)

A 7-year-old girl was admitted to our hospital on May 16, 2019 with chief complaints of pale complexion persisting for 1 year and a cough for 20 days. She presented cough, purulent sputum, occasional chest pain, and the prophylactic use of voriconazole. She was previously diagnosed with IDA at the local hospital because of dizziness and complaints of abdominal pain at presentation on April 2018. On Feb 25, 2019 she was diagnosed with IPH at our hospital based on the manifestation of diffuse fine granular shadows in both lungs on chest computed tomography (CT), the presence of hemosiderin-laden macrophages in the gastric juice, aggravation of her pale complexion, apathy, poor appetite, occasional emesis of gastric contents without bile and blood, absence of hematuria, and presence of black stool (Fig. 1a). From then on, uninterrupted doses of corticosteroids (methylprednisolone, 2 mg/kg·day for 20 days, then 1.54 mg/kg·day for 2 months) were administered. Prior to admission, the CT showed diffuse granular shadows in both lungs and round nodular high-density shadows in the right lung on May 2nd, 2019 (Fig. 1b).The patient is the second child of a healthy couple, and her 16-year-old sister was healthy.

Physical examination revealed a blood pressure of 111/68 mmHg, a respiratory rate of 31 beats/min, a pulse of 100 beats/min and a weight of 31.5 kg. Pale complexion and a fat round face appearance were noted. The patient had a paroxysmal continuous cough, mild coarse breath sounds on auscultation, the liver located 2 cm below the ribs, and the spleen not palpable below the costal region. Moist rales, wheezing, hemoptysis, chest pain, and dyspnea were absent. Neurological examinations revealed no apparent abnormalities. There was no rash, no clubbed fingers, and no bruises on the lips and extremities.

**Fig. 2:**
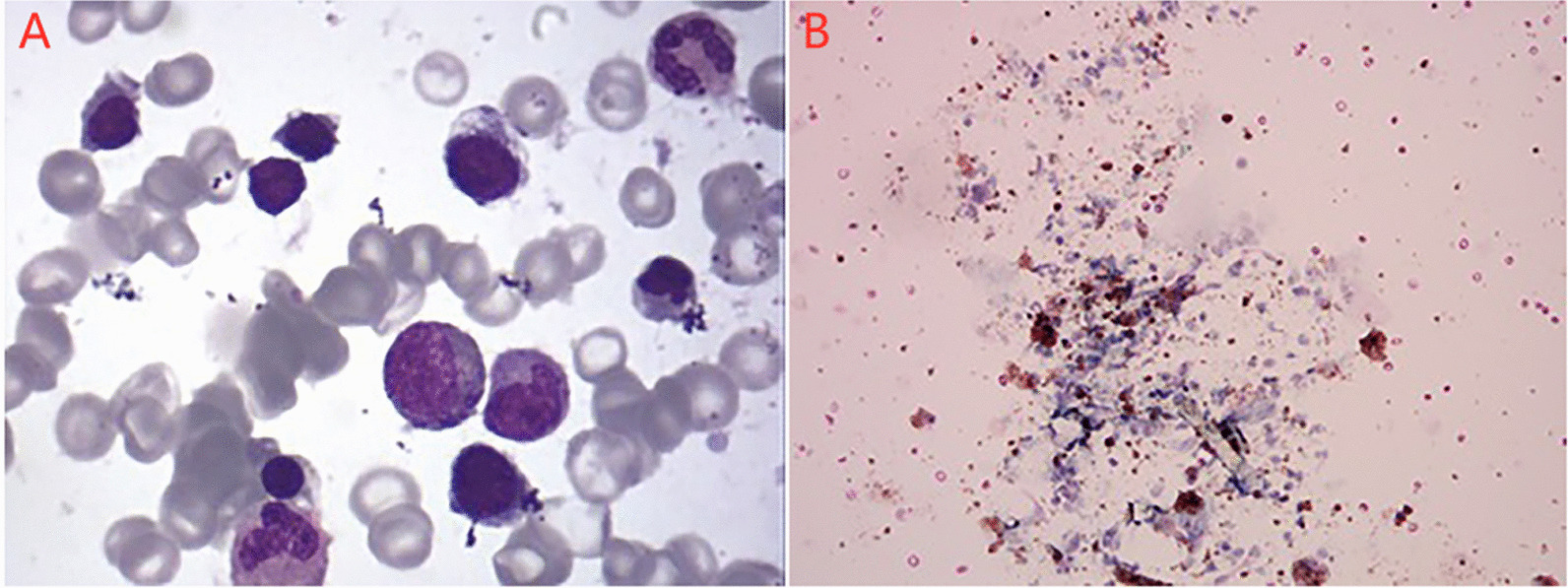
Pathological analysis. **a** Active erythroid hyperplasia, primarily in the late juvenile erythrocytes, was observed and the central globus pallidus of some mature erythrocytes was enlarged. **b** Multiple erythrocytes and large numbers of hemosiderin-laden macrophages were observed

Laboratory investigations revealed a white blood cell count of 20.62 × 10^9^/L (reference range, 4–10 × 10^9^/L), platelet count of 310 × 10^9^/L (reference range, 100–400 × 10^9^/L), hemoglobin level of 152 g/L (reference range, 110–155 g/L), mean erythrocyte volume of 85.3 fL (reference range, 75–92 fL), average hemoglobin content of 25.4 pg (reference range, 26–31 pg), and reticulocyte 1.36% (reference range, 0.5–1.5%). IgG, IgA and IgM were 5.40 g/L, 0.81 g/L and 1.87 g/L, respectively. The right lung contained scattered, circular lesions, and the boundaries were clear on chest CT performed on the day of admission (Fig. 1c, d). Bone marrow biopsy revealed active erythroid hyperplasia, primarily in the late juvenile erythrocytes, and the central globus pallidus of some mature erythrocytes was enlarged, which was consistent with the myelogram of iron- deficiency anemia (Fig. 2a). The anti-tuberculous antibody test, enzyme-linked immunospot assay, tuberculin test, HIV Ig, ANA, P-ANCA, C-ANCA, MPO + PR3, ESR and anti-GBM were negative. Viral nasopharyngeal swab tests, test for *Mycoplasma pneumonia* RNA in the sputum, antibody tests for *Mycoplasma pneumonia* and *Chlamydia pneumonia*, 16S rRNA screening for the presence of gram-positive and gram-negative bacteria, and multiple blood cultures (e.g. for the detection of L form bacteria, anaerobic bacteria, fungus etc.) were negative.

For further pathogen analysis, fiberoptic bronchoscopy was performed on the third day of admission. The lymphocyte, neutrophil, and macrophage counts in the bronchoalveolar lavage fluid (BALF) were approximately 3, 2, and 95%, respectively. Eosinophils were absent. Multiple erythrocytes and large numbers of hemosiderin-laden macrophages were observed (Fig. 2b). The BALF culture was negative. Next-generation sequencing (NGS) (BGISEQ-50) was performed to detect the presence of pathogens in the BALF. The sequencing results were compared to the sequences of pathogens in the four Microbial Genome Databases, including 3446 species of bacteria, 206 species of fungi, 4152 viruses, and 140 species of parasites. A 96% match was found with the reference sequence of *N. abscessus*.

**Fig. 3:**
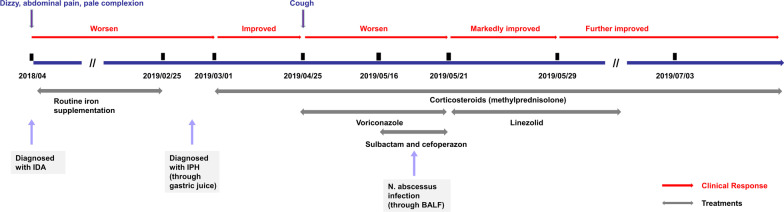
Diagrammatic representation of the treatment and outcome

Sulbactam and cefoperazone were used intravenously. The galactomannan test and (1, 3) β-D glucan test suggested fungal infection, and oral voriconazole was administered as antibacterial and antifungal treatment at the second day after admission. However, no improvement was noted. The lesion in the inferior lobe of right lung was better, cavity formation was observed, new lesions appeared in the left lung, and bilateral pleural thickening was observed (Fig. 1e, f) on May 29, 2019 (14th day of admission). After N. abscessus infection was diagnosed on May 21, 2019, linezolid with a dose of 0.04 g/kg·day was administered (Fig. 3). Finally, the patient was discharged on June 3nd, 2019.

**Fig. 4:**
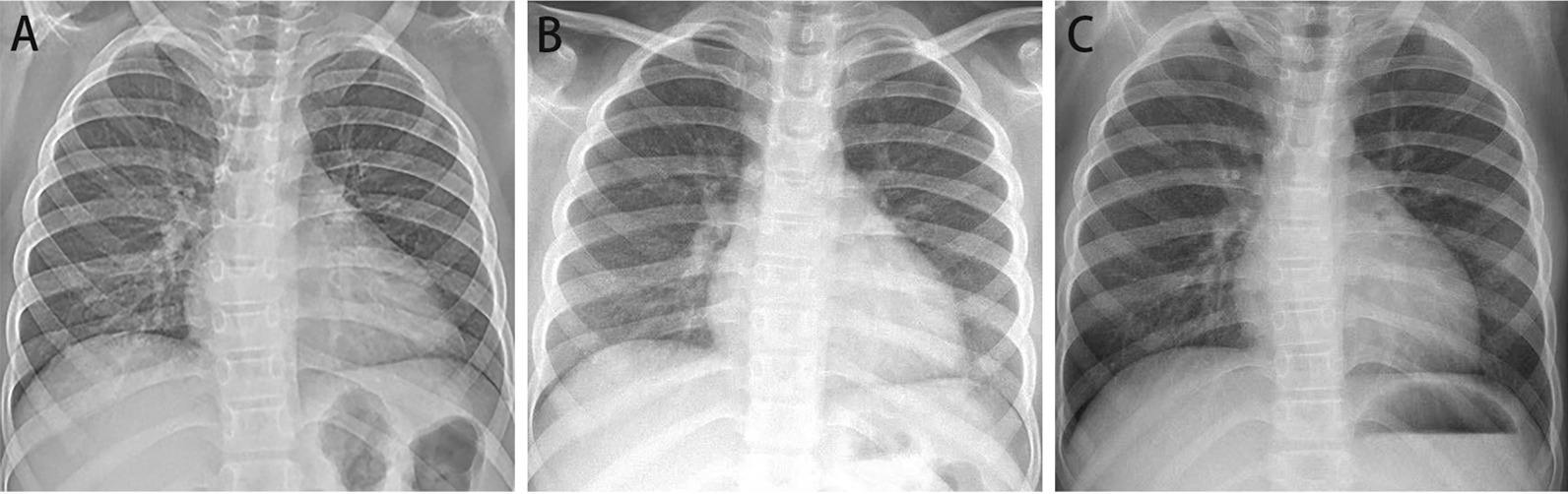
Chest radiography performed at 1, 15 and 21 months after discharge. The pulmonary lesions were significantly improved

Chest radiography performed at 1, 15 and 21 months after discharge (Fig. 4) indicated significant improvement of the pulmonary lesions compared to that at the time of admission with a small dose of methylprednisolone (0.25 mg/kg·day). Oral linezolid with a dose of 0.03 g/kg·day for 7 days was discontinued one week after discharge. The patient’s symptoms and pulmonary lesions had recovered, and the IPH appeared to be well-controlled (Fig. 1g, h).

## Discussion and conclusions

IPH is a pulmonary hemorrhagic disease with an unknown pathogenetic mechanism [6, 7]. Although our patient presented some typical manifestations, including cough, hemoptysis, IDA and pulmonary infiltrating shadows, she was misdiagnosed with IDA until hemosiderin-laden macrophages in gastric juice were noted. Hence, in IDA patients with recurrent cough, IPH should be considered. At present, the risk factors and pathogenesis of IPH remain an enigma. Several risk factors have been proposed with varying degrees of supporting evidence: genetic predisposition, environmental exposure, immunologic association and allergic reaction. According to the relevant examination after admission, the patient did not have cow’s milk protein allergy, and similar diseases were absent among family members. We considered that the pathogenic factors were largely from the environmental exposure and immunologic association owing to the presence of fungal infection and the response to CS.

CS is the main therapeutic strategy [8], which reduces the morbidity and mortality of patients with acute onset alveolar hemorrhage and controls the progression of pulmonary fibrosis [9]. In addition, studies have reported that when prednisolone is used for induction and to maintain remission, early addition of HCQ plus azathioprine/cyclophosphamide may reduce disease flare and steroid toxicity without serious adverse effects [10]. IDA and pulmonary symptoms of our patient improved after CS treatment until 20 days before admission. She presented respiratory symptoms again. Sulbactam, cefoperazone, and voriconazole did not improve the symptoms. NGS of the BALF sample indicated an *N. abscessus* infection. Moreover, linezolid was effective for our patient. These confirmed the diagnosis of Nocardia infection in our patient and suggested that in IPH patients with respiratory manifestations, especially patients receiving CS therapy, a conditioned pathogen (e.g., Nocardia) infection should be considered except fungal infection.

Pulmonary *Nocardia* infection is clinically rare, and most of these infections are serious [11]. The lung is the most common infection site [12]. Both activated macrophages and immunologically specific T lymphocytes constitute the major mechanisms for host resistance to Nocardia infection; so immunodeficiency patients (e.g., long-term CS administration and human immunodeficiency virus infection) are prone to infection with this type of bacteria. However, it also can occur in individuals with normal immune function, particularly in patients with structural abnormalities of the lung, such as bronchiectasis and pulmonary cystic fibrosis [13]. These preexisting structural abnormalities cause respiratory immune defense system dysfunction and can facilitate lower respiratory tract infections and airway colonization. Our patient had a risk factor, long-term CS administration. However, the systemic immune indexes (e.g., immunoglobulin, ANA, P-ANCA, C-ANCA) were all in normal range. Whether IPH also reduces the respiratory local immune defense requires further study.

Pulmonary *Nocardia* may lead to acute necrotic inflammatory responses and presents nonspecific clinical manifestations, including lobar pneumonia, lung abscess, or pulmonary nodular symptoms, similar to the symptoms of a fungal infection [14]. The main symptoms of pulmonary *Nocardia* are fever, cough, initial dry cough, gradual occurrence of purulent sputum or hemoptysis, and severe dyspnea [15]. Our patient presented cough, purulent sputum, and occasional chest pain that did not improve despite the prophylactic use of oral voriconazole for 20 days. Radiological imaging of pulmonary *Nocardia* is non-specific as well and is generally focused under the pleura with occasional presence of pulmonary consolidation, pulmonary infiltration, solitary or multiple nodules, and other changes and with easy formation of voids [16]. These symptoms and radiological imaging provided poor clues to distinguish Nocardia infection from infection by other pathogens (e.g., pulmonary actinomycosis, tuberculosis, fungus), vasculitis or malignant tumors. Moreover, culture of actinomycosis, tuberculosis, fungus and Nocardia is difficult and time-consuming. Fiberoptic bronchoscopy, especially NGS of BLAF may be a recommended method for rapid detection of pathogens.

Although it was regarded that linezolid could not be switched to sulfonamides quickly, our pediatric patient exhibited gradual improvement after linezolid administration and was eventually discharged after 18 days. In the subsequent outpatient follow-up, the clinical symptoms and imaging manifestations of the lungs improved significantly without obvious adverse reactions. Sulfonamides are still used as the first line drug for pulmonary *Nocardia* infection, although in vitro susceptibility results suggest an increased resistance rate of *Nocardia* to sulfanilamide. In addition, aminoglycosides, carbapenes, quinolones and some cephalosporins are sensitive to *Nocardia* and can be used alone or in combination. Linezolid is effective against multi-drug resistant *Nocardia* and can be used to treat resistant strains [17].

In conclusion, Nocardia infection should be considered in the differential diagnoses for IPH patients receiving CS therapy, especially in patients with “pneumonia” who fail to respond to conventional empirical antibiotic therapy. NGS of BLAF may be used to quickly identify the Nocardia. Sulfonamides or linezolid are effective for treating pediatric pulmonary Nocardia infection.

## Data Availability

All data generated or analyzed during this study are included in this published article [and its supplementary information files].

## Abbreviations

IPH: Idiopathic pulmonary hemosiderosis
*N. abscessus*: *Nocardia abscessus*
CS: Corticosteroid
NGS: Next-generation sequencing
BALF: Bronchoalveolar lavage fluid
IDA: Iron-deficiency anemia
Ig: Immunoglobulins
HIV: Human immunodeficiency virus
CT: Computer tomography
